# Improving Tracking of Trajectories through Tracking Rate Regulation: Application to UAVs

**DOI:** 10.3390/s22249795

**Published:** 2022-12-13

**Authors:** Fernando Diaz-del-Rio, Pablo Sanchez-Cuevas, Pablo Iñigo-Blasco, J. L. Sevillano-Ramos

**Affiliations:** ETS Ingeniería Informática, Universidad de Sevilla, Av. Reina Mercedes s/n, 41012 Sevilla, Spain

**Keywords:** UAV, mobile robots, path following, trajectory tracking, error adaptive tracking, Lyapunov stability theory

## Abstract

The tracking problem (that is, how to follow a previously memorized path) is one of the most important problems in mobile robots. Several methods can be formulated depending on the way the robot state is related to the path. “Trajectory tracking” is the most common method, with the controller aiming to move the robot toward a moving target point, like in a real-time servosystem. In the case of complex systems or systems under perturbations or unmodeled effects, such as UAVs (Unmanned Aerial Vehicles), other tracking methods can offer additional benefits. In this paper, methods that consider the dynamics of the path’s descriptor parameter (which can be called “error adaptive tracking”) are contrasted with trajectory tracking. A formal description of tracking methods is first presented, showing that two types of error adaptive tracking can be used with the same controller in any system. Then, it is shown that the selection of an appropriate tracking rate improves error convergence and robustness for a UAV system, which is illustrated by simulation experiments. It is concluded that error adaptive tracking methods outperform trajectory tracking ones, producing a faster and more robust convergence tracking, while preserving, if required, the same tracking rate when convergence is achieved.

## 1. Introduction

In a state space system, all the possible internal states of the system can be represented as a vector of variables. Typical control engineering problems in these systems are the stabilization problem, i.e., how to take the system to a fixed point in its state space, and the *tracking* problem, i.e., how to follow a desired trajectory or path. This tracking problem has been profusely studied in the area of motion control of mobile robots and autonomous vehicles, where the desired path is either memorized or previously generated [1,2].

In the particular case of UAVs (Unmanned Aerial Vehicles), it must be remarked that paths are usually defined as a set of straight lines and circular-orbit paths connecting several waypoints. This means that these paths usually contain singular points in the intersections of these lines, that is, they are not feasible trajectories for the UAV itself, but imprecise paths that the UAV cannot accurately track. Nonetheless, a convenient interpolation can convert this piecewise path into a smooth UAV trajectory passing over the desired waypoints, which should fulfill its own state equations. Note that having well-defined feasible desired trajectories is important when using UAVs safely (e.g., avoiding collisions) in many applications, such as multi-UAV systems, cluttered urban environments, etc. Navigation sensors are usually integrated into the robot in order to determine its current position and, thus, calculate the errors between the desired and actual trajectory.

**Figure 1:**
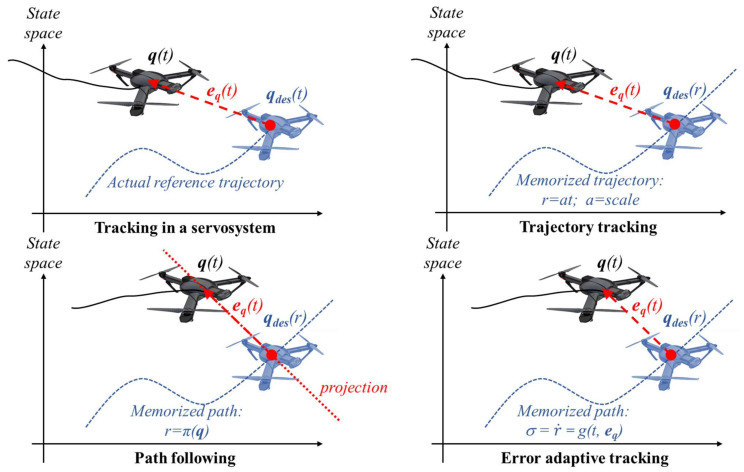
Classification of tracking methods regarding the descriptor parameter.

The most common tracking method is called “*trajectory tracking*” (TT) or “*reference tracking*” and it explicitly considers time in the tracking [1]. In this case, the controller aims to bring the robot as near as possible to a moving target (or *reference*) point (Figure 1, top right). It is like servosystems (Figure 1, top left) where it must be guaranteed that the system will approach the desired point in a deterministic time. Examples of this kind of tracking can be found in most industrial robot applications (due to their strict real time characteristics). In mobile robots, pursuing a real moving objective (such as an antimissile system) is an example of a task that needs time determinism.

The second group, usually called “*path following*” (PF) (Figure 1, bottom left), does not consider timing requirements and simply tries to converge to a path. A reference point on the path must be selected at each instant according to some relation between actual robot state and path shape, e.g., the “closest” point to the robot’s position. Consequently, a notable PF inconvenience is assuring the projection uniqueness for all possible paths.

A common example of PF is car driving, which can be extended to most Intelligent Transportation Systems (ITS) applications. For instance, in cars, usual control approaches select a point at a look-ahead distance on the road and the vehicle is driven to that point. Linear speed is preset, while orientation (or steering) is the single control variable used to perform the convergence.

However, there is confusion in the literature regarding the terminology used for these methods. For instance, a tracking rate that adapts to system errors has been used in [3] to improve the TT guidance results for underactuated vehicles in the presence of parametric modeling uncertainties, although these authors use the term ‘path following’ to refer to their implementation. There are other approaches, such as the one inspired by the Dynamic Time Warping (DTW) algorithm (studied extensively in the automatic speech recognition literature) in [4], where a strictly increasing rate of progression ($\dot{r}$ > 0) is selected by minimizing a cost function for finite-duration movements.

Although we can find several excellent compendia of both methods in some classic books [1], the question of which tracking method is the most adequate for a given application is an active research area and many papers choose to implement a TT or a PF controller for UAVs and other mobile robots, depending on the application or with the purpose of easing the finding of a suitable controller (see more references in context in the rest of this section). This question has been elucidated for some simple paths and specific systems: in [5], TT and PF controllers were investigated for a linear time-invariant system with unstable zero dynamics, and it was demonstrated (for the simple PF task of moving the vehicle along a straight line) that there is a fundamental performance limitation for TT, which does not appear for PF method.

In the field of UAVs, many variants have appeared, which are called “guidance laws”, and are actually based on TT or PF methods. Many of them use a virtual target point (VTP) on the path, which is selected through some projection, such as the line-of-sight (LOS) point situated at a certain look-ahead distance from the nearest path point to the robot. The selection of this point implies that they are a PF variant. Among these methods, we find a first set that uses simple and intuitive methods, such as the classic carrot-chasing algorithms and the Pure Pursuit. The number of works that have used these simple methods for UAVs is considerable, with [6,7] being perhaps the first ones.

There is another set of methods that select the projection point using a pair of circles that intersect with the desired path, which have been named “nonlinear guidance laws” (NLGL). These methods were used many years ago [8], and are still very common in recent years [9].

An alternative to guidance laws, which appeared 15 years ago, are those based on vector fields (VFs). A VF is built for each position in the state space and as a function of each specific path, that is, it is a geometric approach that computes a special projection that returns a vector. This vector defines some of the desired variables that the system must follow. Thus, according to our classification, VF are also a type of PF. It is worth mentioning that, up to date, not all state variables are determined by the VF, and the rest of the variables that remain free must be calculated by the controller. In this respect, a Lyapunov-based controller can be simplified because some of the desired states are predefined by the VF [10,11].

To sum up, designing VFs in 3D is not simple, and requires significant work [12]. Maybe the first proposal of a VF-based PF algorithm was developed in [13], as an intuitive and easy way to compute the desired heading angle for simple paths, such as straight lines and circles. Many other VFs for specific paths have followed since then, such as [14,15,16]. No VF has been implemented yet for any generic path; hence, this method should evidently come across the same drawbacks as PF. It is not guaranteed that the virtual field exists for a generic path, even for a simple one, such as a pure rotation around the robot center of mass.

In TT, time is the usual descriptor parameter of a path. Since time is an intuitive parameter, TT seems to be the most straightforward method. However, for the rest of the approaches, other path-descriptor parameters are possible. For example, in differential geometry, the natural arc parameter, which makes the linear speed equal to one, is generally preferred. In this paper, for the sake of generality, the descriptor parameter is denoted by *r*. Therefore, other groups of tracking methods can be defined to explicitly control the progression rate of a moving virtual target to be tracked; i.e., they impose a pace for *r* or a value for $\dot{r}$ (derivative with respect to time). Equivalently, in these methods, the real robot is forced to follow a virtual robot (also called “reference” robot) that goes along the reference path at a variable pace, which may be null when necessary; i.e., the reference robot can “wait” for the real one [17,18]. This pace can be selected with several purposes (as shown below).

Some scattered examples can be found in the literature, where the explicit control of progression of the “virtual target” (that is, the VTP) helps design a control law. For instance, in [18] a complete practical application, where the motion of the descriptor parameter was governed by a differential equation depending on the errors’ and path’s shape, was developed. In [19], a term related to the curvature (called “curvature effort” in that work) was defined, and a penalty factor based on the curvature effort was introduced in the dynamics of the path’s description parameter to prevent the performance degradation of the tracking when the dynamic and kinematic constraints are exceeded. In [20], the target progression was tailored to design a nonlinear adaptive control law, which yields the convergence of the (closed-loop system) error trajectories to zero in the presence of parametric modelling uncertainties.

This family of methods can be considered a different path-tracking method that can be named *error adaptive tracking* (EAT) [13] (Figure 1, bottom right). Furthermore, EAT methods can be divided into two categories, depending on whether time deterministic following is expected or not. Basic EAT variants can be named “non-deterministic” EAT (NDEAT) because no aspect of time determinism is pursued. On the other hand, tracking rate adaptation to system errors can be combined with convergence of *r* to time (that is, convergence to the TT method). In this case, the rate of *r* can be extended to include the “inaccuracy in the deterministic tracking”, i.e., the difference between the descriptor parameter *r* and time *t*. For this reason, this variant can be named “soft” deterministic error adaptive tracking (SDEAT) [21]. A tailored control law that includes a variable tracking (similar to that of SDEAT) of the virtual target that helps design the control law was exploited in [22] (these authors called it path tracking).

The aim of this paper is to provide a formal description and generalization of the EAT tracking method (which was used in particular cases of terrestrial and underwater vehicles [21]), and to show how it can be used *in any system using the same control law*, with the additional advantage of improving error convergence and robustness. Afterwards, EAT method is applied to a UAV model to show its benefits. We must emphasize that this paper is not focused on the design of new control laws. The selection of a tracking method, or more specifically, a proper form for $\dot{r}$ when using EAT, has been exploited in some systems [3,20,22] with the aim of finding a stabilizing control law (mainly via second Lyapunov method). On the contrary, the present study is focused on how the EAT method (instead of TT) can be applied using the same controller just by selecting a proper pace for $\dot{r}$, having the additional benefits that error convergence and robustness are improved. As a result, the burden of finding a new controller or a special path projection (see VF, carrot-chasing and so on in the Introduction section) will not be necessary. An additional benefit is that the switching between these two tracking methods (TT and EAT) can be performed smoothly, since the controller is exactly the same. Our approach is here particularized for a UAV, but it is worth mentioning that the same procedure can be applied to other non-linear systems. In fact, this is the first time (to the best of our knowledge) that an EAT method has been applied to a UAV, despite the fact that many tracking methods have been implemented for UAVs (see Introduction).

In order to understand properly the different tracking methods and the notation used in this work, we present first the simple case of a one-state system: $\dot{x}=u$, where *x* is the coordinate and *u* is the input. The goal is to follow a reference *x_des_={x_des_(r)}* made by a virtual system, which must fulfill $x_{des}^{’}=u_{des}\left(r\right)$, where (’) holds for derivative with respect to *r*. Let us suppose that the whole reference path is known (memorized) and *r* is extended all over real line R. Thus, the following relations for *r* hold: *u_des_(t)=* $\dot{r}$ *u_des_(r)*, and ${\dot{x}}_{des}=u_{des}\left(t\right)\;$*=* $\dot{r}$ *u_des_(r)*.

This one-coordinate system will permit us to easily extract and analyze the differential equations implicated in this proposal and to clearly see EAT running. Moreover, we will be able to gain insight of the influence of tracking rate election over system behavior. Note that the concept of trajectory does not exist for a coordinate only, but our analysis and conclusions can be extended to a system with several coordinates.

For a fair comparison between TT and EAT, we will use the same control law for both methods. We briefly present a problem that affects the model considered by the controller (which are usual for UAVs). Evidently, with no perturbation, big errors or unmodeled effects stationary tracking would be perfect and, consequently, there would be no need for studying improvements introduced via tracking rate changes. The main idea is that, using the EAT method, problems that affect the tracking will be partially “absorbed” by the tracking rate in order to reduce tracking errors and to improve convergence.

If TT were applied, the system error would be merely the difference: *e(t) = x(t) -x_des_(t)*, and the state equation for the error would be: $\dot{e}$*(t) = u(t) – u_des_(t);*

It is clear that a simple convergent control law is: *u(t)= u_des_(t)–K_p_e , K_p_ >0*

This yields the TT error equation: $\dot{e}$*(t)= -K_p_e.*

The solution of the previous equation is: *e(t) = e(0) exp(-K_p_t),* where *e(0)* is the initial error. Therefore, exponential convergence is ensured, with a characteristic time constant t = (*K_p_*)^−1^.

**Figure 2:**
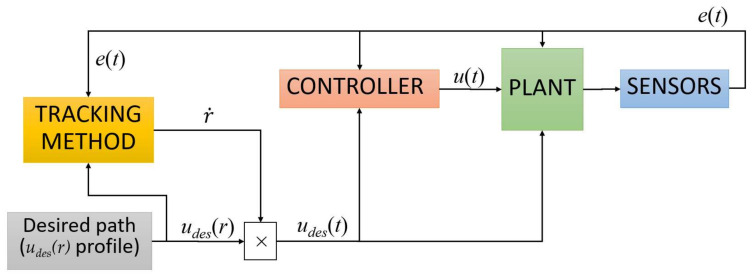
Diagram block of the control of a plant that considers the descriptor parameter evolution.

Figure 2 depicts the role that the tracking methods play in the feedback control of a simple one-state plant. Common blocks for the plant, controller and sensors work as usual. However, the desired trajectory sent to the controller is computed through the product of the desired path profile *u_des_(r)* and the $\dot{r}$ evolution, which is selected by the desired tracking method.

On the other hand, if EAT were to be applied, the system error (a superscript *r* is added to clearly distinguish this error definition from that of TT) would be: *e^r^(t) = x(t) − x_des_(r(t)),* and the error equation: $\dot{e}$*^r^(t)= u(t) −* $\dot{r}$*u_des_(r)*. Now the same simple control law yields:

*ė*^*r*^= −*K*_*p*_*e*^*r*^ + *ṙu*_*des*_(*r*) − *u*_*des*_(*r*);

An intuitive proposal for a NDEAT tracking rate can be (this intuitive form fulfills completely the mathematical condition given by the Lemma in the next section):

$$\dot{r}=1+\frac{K_{r}}{u_{des}}e_{}^{r};K_{r}>0$$

This yields to the EAT error equation: $\dot{e}$*^r^(t) = -K_p_e^r^ − K_r_e^r^*. The error evolution includes a new parameter *K_r_* that considers the rate of tracking, which is: *e(t) = e(0) exp((−K_p_ − K_r_ )t),* where *e(0)* is the initial error. Exponential convergence for EAT method has now the characteristic time constant t = (*K_p_ + K_r_*)^−1^, which is faster than that of TT because error decreasing is produced by two causes: the control law and the tracking rate selection.

Likewise, if SDEAT were to be applied, system error could be defined as: *e^rt^(t) = x(t) − x_des_(r(t)) + A_rt_(t − r), A_rt_* >0. Therefore, an intuitive proposal for SDEAT tracking rate could be:

$$\dot{r}=1+\frac{K_{rt}}{A_{rt}+u_{des}}e_{}^{rt}\;\;K_{r}t>0$$

**Figure 3:**
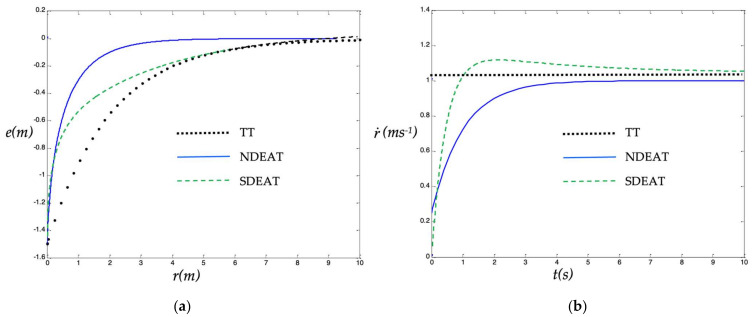
(**a**) TT, NDEAT and SDEAT transient behaviors for a big initial error. (**b**) $\dot{r}$ evolution.

Transient behavior is more interesting with respect to the advancement along the desired path *r* because this will be the main objective when following a memorized path, for example, in mobile robot applications. Previous equations were simulated using MATLAB, with the following conditions: simulation time = 10 s, *e(0)* = −1.5 m, *K_p_* = 0.5 s^−1^ , *K_r_* = 0.5 s^−1^ , and reference path defined by *u_des_(r)* = 1 m/s. In Figure 3a we represent TT and NDEAT error behaviors as a function of parameter *r* (being *r = t* for TT). Likewise, $\dot{r}$ evolution is shown in Figure 3b.

The most interesting fact in Figure 3b happens during the first transitory moments for EAT methods. Here, $\dot{r}$ gets low values, thus, the desired point *x_des_(r)* “waits” for the robot to approach it. For a system with several coordinates, this means that a faster convergence to the desired path can be reached with the EAT method. At the extreme case, if errors were big, this approximation would become a straight line to the reference *x_des_(r(t))*. This desirable behavior clearly resembles that of the PF method [1,5].

On the contrary, for TT we find the usual behavior of a tracking system; the objective advances continuously pulling the system ahead. In this way, in a system with several coordinates, this attraction will prevent the system from approaching the path, and so the system convergence will be delayed in relation to the path parameter *r*. Note also that the final value of *r* for NDEAT in Figure 3b is lower than the final value of *r* (or *t*) for TT because $\dot{r}$ takes small values for the first seconds.

In the case of SDEAT, it can be seen that it behaves similar to NDEAT during the first moments when errors are big (Figure 3a). That is, $\dot{r}$ stays low (Figure 3b) because the system intends to approach point *x_des_(r)* (*r* is almost constant). Therefore, we will achieve a fast convergence to the path (in case of several coordinates). However, when the system is approaching convergence, $\dot{r}$ grows above one, and the system tries to be deterministic by tracking the reference point *x_des_(t)*, that is, the system recovers real-time conditions. In this last part, the system velocity continues to be slightly bigger than the reference velocity *u_des_(r)* to reduce the difference *r−t*. SDEAT evolution of Figure 3 has been reproduced with exactly the same conditions and control law. Constants for SDEAT were chosen as *A_rt_ = 2.0* ms^−1^*, K_rt_ = 2.0* s^−1^.

In addition, let us analyze the existence of a parametric modeling uncertainty. Imagine that the real system equation has a *δ* deviation from the ideal model, that is, the real system behaves actually as $\dot{x}=\left(1+\delta \right)u$. Applying the same control law, the error equation becomes now:

*ė*(*t*)= −*K_p_*(*1*+*δ*)*e* + (*d* + *1* − *ṙ*) *u*_*des*_(*r*)

Therefore, a steady error *e_ss_* is unavoidably present. In the case of TT ($\dot{r}$ *= 1*), when the stationary state is reached ($\dot{e}$→0), we arrive at

$$e_{ss,TT}=\frac{\delta u_{des}}{\left(1+\delta \right)K_{p}}$$

However, this error would be scaled down by $\dot{r}$ if NDEAT were used. Applying the NDEAT proposal for $\dot{r}$, the stationary state can be easily found as:

$$e_{ss,NDEAT}=\frac{\delta u_{des}}{\left(1+\delta \right)K_{p}+K_{r}}$$

It is clear that *e_ss,NDEAT_* becomes smaller than *e_ss,TT_*, as the reduction in tracking rate (that is, $\dot{r}$ < 1 for NDEAT) is absorbing the unmodeled behavior. More exactly for constants *K_p_ = K_r_* = 0.5 s^−1^, the stationary error is divided by almost 50%.

To sum up, two important considerations must be taken into account:

- As the control law and the system become more complex, the design of tracking rate $\dot{r}$ should contemplate more circumstances as it is discussed below for the case of UAVs.
- For complex systems, it is obviously more difficult to find robust control laws that behave well enough under several problems (such as unmodeled behaviors, motor delay responses and so on). When this happens, the EAT method may provide a new form of avoiding oscillations, divergences, error enlargements, etc. This will be presented at the following sections, where a generic method is formulated to extend the EAT for any controller that is based on a Lyapunov function.

The paper is organized as follows. Basic formulation and the lemmas to use EAT methods from a Lyapunov-based controller are stated in Section 2. In Section 3, an application case is discussed in depth, a UAV model. Its asymptotically stable control law is considered, and its validity for various EAT methods is demonstrated. Evaluation of these methods through simulation is discussed to illustrate the EAT benefits in Section 4. Finally, conclusions are presented in the last section.

## 2. Conversion of Trajectory Tracking into Error Adaptive Tracking for Lyapunov-Based Controllers

In this section, we present a theoretical formulation of the tracking equations and some Lemmas for using EAT straightforwardly from a Lyapunov-based controller. Let us consider the state space representation of a dynamic system model: $\dot{q}=f_{q}\left(q,u,t\right)$, where ***q*** is the state vector of dimension *m*, ***u*** is the input vector of dimension *n*, and *t* is time. The initial conditions are given by ***q***(0). A *memorized*, *reference or desired* path or trajectory (or merely *path*) ***q_des_***(*r*) can be described by a single-descriptor parameter, namely *r*. This path should be covered by the system, that is, it must fulfill the system model: $q_{des}^{’}=f_{q}\left(q_{des},u_{des},r\right)$, where (´) denotes derivative with respect to *r*. As we are interested in convergence to a path, we assume in this work that the desired trajectory has no end and it never stops, that is, *r*є(-∞, ∞), $q_{des}^{’}\neq 0,\forall r$ (because if it ended at a certain point, this would be a problem of stabilization instead of tracking).

To study the tracking, an error state vector should be defined through a diffeomorphism ***e*** = *h_e_*(***q***, *r*, *t*), such that ***e*** = 0 if and only if ***q***(*t*) = ***q_des_***(*r*(*t*)). Note that *r* dependence can now be introduced in the definition of error ***e***. Likewise, the input vector may be redefined as ***v*** = *h_v_*(***q***, *r*, ***u***, *t*) in order to express the dynamics in a more convenient form. In practical cases, the dependence of error and input vectors on *r* are usually through ***q_des_****(r)* and ***u_des_****(r)*, which are merely functions of *r*. Therefore, a new state variable *r* appears, whose state equation can be freely defined (“modeled”) in general as: $\dot{r}=\sigma \left(e,r,t\right)$, where σ can be considered a new input, so dependence on **v** is prevented in σ. Thus, the system error model is now $\dot{e}=f\left(e,r,v,\sigma ,t\right)$, and the initial conditions are given by ***e***(0), *r*(0).

Minimal tracking control objectives for these state variables {***e***, *r*} can be set to

*e→0* when *t→∞* (1a)

|*ṙ*| bounded ∀t, *r*→∞ when *t*→∞ (1b)

Objective (1b) is needed to prevent *r* from jumping suddenly, so tracking is performed smoothly. These objectives are to be satisfied through the proper selection of control laws:

*v* = *c_v_*(*e*, *r*, *t*) (2a)

*σ* = *g*(***e***, *r*, *t*). (2b)

We want to point out that the expression *g*(***e***, *r, t*) is just a control law for *r*, and the behavior of *r* (or its rate of progression) can be designed for any application—still preserving the objectives stated in (1b).

A trivial rate of progression can be performed just by identifying parameter *r* with time, that is, *r*(*t*) *= t* or σ=1, which yield the simplest TT. This would mean that ***q_des_***(*r*) advanced continuously pulling the system forward. In this case, error coordinates can be simply defined as ***e_q_***(*t*) ***= q****(t)****-q_des_***(*t*). Nevertheless, TT can be extended with a more general assumption: let parameter *r* be a strict increasing function of time to fulfill objectives in (1b), for example, *r = at, a > 0*. Therefore, error coordinates can be ***e_q_****(t) **= q**(t)****-q_des_***(*r*(*t*)).

However, if time was not critical, the tracking methodology could be freely designed, as the whole trajectory is known a priori. The most common alternative in the mobile robotics literature is *path following*. This is based on some relation between the actual system state and the whole path. This relation or *projection* will give us the desired point ***q_des_***(*r*), i.e., the descriptor parameter *r* as a function of the actual position and the path. Differently from the TT case, here the real system must aim to follow this point. For example, the desired point is usually selected to be the point on the path that is “closest” to the actual robot’s position [23,24]. The main drawback of PF is that (to the authors’ knowledge) projection uniqueness has not been guaranteed for all possible paths ***q_des_***(*r*). This problem may occur when the projection is fulfilled for an interval of *r* [23], which means that (1b) cannot be satisfied (*r* is undetermined in this interval). Finally, we have the EAT method, which has very interesting properties: it can be applied to every tracking system and to all sets of paths (because it does not depend on any projection); it can also consider timing requirements, and, as it will be discussed in the next section, its controller design can be performed in a way similar to that of TT, but achieving faster convergence and higher robustness. In the approach presented here, *the same control law* can be used for TT and EAT methods, thus, facilitating the design of the controller and allowing to choose between TT and EAT when needed.

It can be noted that PF on the one hand, and TT or EAT on the other, must define completely dissimilar control laws. For example, in a system with two error variables and two inputs, the PF projection causes a constraint that eliminates one of the variables [23], which implies that PF control law is applied only to one variable. A common approach consists of setting one of the inputs to be constant, thus, forcing the system to move. Another approach consists of selecting a relation for both inputs that implies the overall input to never be null. This “motion exigency” is not present in EAT (or TT). Depending on the regulations performed for both tracking methods, one method will present potential benefits over the other or vice versa. On the other hand, the control law will be identical when applying NDEAT or SDEAT (see Lemmas below), and no design or tuning of a new controller is needed.

Moreover, when using EAT, the convergence will be, at least, as fast as that obtained with TT. In the next sections, this general procedure is illustrated for a PVTOL (planar vertical take-off and landing aircraft). The subsequent tests will demonstrate that *a)* EAT presents a faster convergence than TT, and *b)* it is valid even for those paths where PF cannot be applied. Nonetheless, as mentioned before, the procedure shown here can be applied to any other system that uses a Lyapunov-based control law.

Before presenting the Lemmas, it should be pointed out that their objective is to change the tracking from TT to EAT due to the good properties of the latter. As a direct consequence of these Lemmas, when using EAT the question would be: what tracking rate must be selected for the EAT method so that the same controller presents a faster convergence to the desired posture? In this respect, finding an appropriate tracking rate is crucial for nonholonomic system controllers. This is a direct consequence of the Brockett’s Theorem [25], which prevents the existence of a smooth feedback stabilization control law. We mean that a smooth controller may fail if the tracking rate is different from σ=1 (this is evident if the rate becomes near σ=0, that is, if the tracking tends to stabilization). Another consequence of nonholonomic constraints is that derivatives of a Lyapunov function *V* cannot be a negative definite function but only a negative semidefinite function. This can be observed for those postures where only an error is not null and the movement that should reduce this error is prohibited, such as a lateral displacement in a car.

The next Lemmas allow selecting a valid rate that additionally improves initial TT convergence and introduces an interesting relation with the PF method (see Remark 3).

**Lemma** **1.** *Conversion of TT into NDEAT.*

Let $\dot{q}=f_{q}\left(q,u,t\right)$ be the model of a system that must follow a smooth desired path given by ***q_des_***(*r*), *r*є(−∞,∞), which fulfills $q_{des}^{’}=f_{q}\left(q_{des},u_{des},r\right)$, with ***u_des_***(*r*) ≠ 0 ∀r. Let ***e^t^*** *= h_e_(**q**,**q_des_**(t))* be the definition of TT errors, and ***e^r^*** *= h_e_(**q**,**q_des_**(r(t)))* those of EAT errors, being *h_e_* also smooth. Let us suppose that there exists a positive definite Lyapunov function *V*(**e^t^**), with the intention that a smooth control law ***v =*** *c_v_(**e^t^**, t*) makes $\dot{V}$ be negative semidefinite and uniformly continuous, so it can be proved that ***e^t^***=0 is a global asymptotically stable equilibrium point for the path ***q_des_***(*t*).

With these assumptions and for the same control law evaluated on *r*: ***v =*** *c_v_(**e^r^**, r)*, we have that ***e^r^*** *= h_e_(**q**, **q_des_**(r(t))) = 0* is also a global asymptotically stable equilibrium point, if a uniformly continuous NDEAT rate $\dot{r}=\sigma \left(e^{r},r\right)=1+e_{\sigma }\left(e^{r},r\right)$ is chosen, so that $e_{\sigma }\langle {\left.\frac{\partial V}{\partial e}\right|}_{e=e^{r}},\frac{\partial h_{e}}{\partial q_{des}}f_{q}\left(q_{des},u_{des},r\right)\rangle$ is negative semidefinite with respect to ***e***. Here, ⟨x,y⟩ represents the dot product of ***x, y*** and $\frac{\partial }{\partial x}$ stands for the gradient for a scalar function, or the Jacobian for a vector function.

**Proof** **of** **Lemma** **1.** Using the chain law, ${\dot{q}}_{des}=\sigma \;q_{des}^{’}={\sigma \;f}_{q}\left(q_{des},u_{des},r\right)={\sigma \;f}_{q,\mathrm{des}}$, where it has been called $f_{q,\mathrm{des}}=f_{q}\left(q_{des},u_{des},r\right)$ for clarity purposes. Deriving **e^r^** = h_e_(**q**,**q_des_**(r(t))) and using the change $\sigma =1+e_{\sigma }$,

$${\dot{e}}^{r}=\frac{\partial h_{e}}{\partial q}\dot{q}+\frac{\partial h_{e}}{\partial q_{des}}{\dot{q}}_{des}=\frac{\partial h_{e}}{\partial q}\dot{q}+\frac{\partial h_{e}}{\partial q_{des}}f_{q,des}+e_{\sigma }\frac{\partial h_{e}}{\partial q_{des}}f_{q,des}$$

The previous derivative can be expressed as:

$${\dot{e}}^{r}={\dot{e}}_{t=r}^{t}+e_{\sigma }\frac{\partial h_{e}}{\partial q_{des}}f_{q,des}\;,$$

where ${\dot{e}}_{t=r}^{t}=\dot{q}-q_{des}^{’}\left(t=r\right)$ represents the tracking error rate for the TT case when *t = r*. By computing the derivative of *V*(**e^r^**), we obtain

$$\dot{V}\left(e^{r}\right)=\langle {\left.\frac{\partial V}{\partial e^{}}\right|}_{e=e^{r}},{\dot{e}}^{r}\rangle =\langle {\left.\frac{\partial V}{\partial e^{}}\right|}_{t=r},{\dot{e}}^{r}\rangle =\langle {\left.\frac{\partial V}{\partial e^{}}\right|}_{t=r},{\dot{e}}_{t=r}^{t}\rangle +e_{\sigma }\langle {\left.\frac{\partial V}{\partial e^{}}\right|}_{e=e^{r}},\frac{\partial h_{e}}{\partial q_{des}}f_{q,des}\rangle$$

Using the hypothesis, it holds that $\dot{V}$ is negative (at least) semidefinite and uniformly continuous in time. Therefore, the resulting NDEAT tracking will also make **e^r^** be a globally asymptotically stable equilibrium point for the path ***q_des_***(*r*), as was ***e^t^***. □

**Remark** **1.** *Note that the convergence of the NDEAT is, at least, as fast as that of the TT because the term* $e_{\sigma }\langle {\left.\frac{\partial V}{\partial e}\right|}_{e=e^{r}},\frac{\partial h_{e}}{\partial q_{des}}f_{q,des}\rangle$*decreases or maintains the temporal rate of V*.

**Remark** **2.** *Note that a simple election like* $e_{\sigma }=-K_{\sigma }\langle {\left.\frac{\partial V}{\partial e}\right|}_{e=e^{r}},\frac{\partial h_{e}}{\partial q_{des}}f_{q,des}\rangle \;;\;K_{\sigma }>0$*implies that*$e_{\sigma }\to 0$; σ→1*, i.e., the same desired tracking rate is preserved when the convergence is achieved.*

**Remark** **3.** *Path following controllers usually project real system posture over the reference path by choosing that reference point that minimizes some kind of distance. A common and sensible distance is given by the proper Lyapunov function* [1]*. In fact, this function gives an idea of the amount of error, so the point on the path with minimum errors is chosen. In this case, the projecting point looks for the value of r that makes the derivative of V null for a fixed system state, that is,*

$${\left.\frac{\partial V}{\partial r}\right|}_{q=constant}=0\;;\;{\left.{\left.\frac{\partial V}{\partial e}\right|}_{e=e^{r}}\frac{\partial e}{\partial r}\right|}_{q=constant}=\langle {\left.\frac{\partial V}{\partial e}\right|}_{e=e^{r}},\frac{\partial h_{e}}{\partial q_{des}}f_{q,des}\rangle =0\;;$$

The previous equation zeroes the same term that multiplies to $e_{\sigma }$ in $\dot{V}\left(e^{r}\right)$, which implies that the chosen $e_{\sigma }$ of Remark 2 makes EAT tracking tend to that of PF. Thereby, EAT conserves most of the advantages of the PF method [1,5], while avoiding its main obstacle: the non-uniqueness of the selection of a point in the path when the robot is far from it (in other words, the need for the robot to stay in a tube around the path).

If the SDEAT method were to be applied, a way to obtain the proper function $\sigma =\sigma \left(e^{r},r,t\right)$ is to select the next variant of the Lyapunov function: $V_{2}\left(e^{r},t\right)=V\left(e^{r}\right)+\frac{1}{2}A_{rt}{\left(r-t\right)}^{2}$, *A_rt_*>0. Proceeding correspondingly,

$${\dot{V}}_{2}\left(e^{r},t\right)=\dot{V}+e_{\sigma }A_{rt}\left(r-t\right)=\langle {\left.\frac{\partial V}{\partial e}\right|}_{t=r},{\dot{e}}_{t=r}^{t}\rangle +e_{\sigma }\left({\langle \left.\frac{\partial V}{\partial e}\right|}_{e=e^{r}},\frac{\partial h_{e}}{\partial q_{des}}f_{q,des}\rangle +A_{rt}\left(r-t\right)\right)$$

An evident SDEAT proposal that keeps ${\dot{V}}_{2}$ uniformly continuous in time and negative definite (other σ are possible), is making the last term quadratic by carrying out

$$\sigma =1-K_{\sigma }\left({\langle \left.\frac{\partial V}{\partial e}\right|}_{e=e^{r}},\frac{\partial h_{e}}{\partial q_{des}}f_{q,des}\rangle +A_{rt}\left(r-t\right)\right),\;K_{\sigma }>0$$

This allows us to enunciate the following Lemma.

**Lemma** **2.** *Conversion of TT into SDEAT.*

For the same conditions of Lemma 1, ***e*** *= h_e_(**q**,**q_des_**(r(t))) = 0* is a global asymptotically stable equilibrium point, if a uniformly continuous SDEAT rate $\dot{r}=\sigma \left(e^{r},r\right)=1+e_{\sigma }\left(e^{r},r,t\right)$ is chosen, so that $e_{\sigma }\left({\langle \left.\frac{\partial V}{\partial e}\right|}_{e=e^{r}},\frac{\partial h_{e}}{\partial q_{des}}f_{q,des}\rangle +A_{rt}\left(r-t\right)\right)$ is negative semidefinite with respect to ***e*** and to (*r − t*), with *A_rt_* > 0.

**Proof.**  The proof can be guided in a way similar to that of Lemma 1, using the given form of ${\dot{V}}_{2}\left(e^{r},t\right)$.□

**Remark** **4.** *Note that r tends to t, so TT tracking rate can be achieved in the end.*

Straightforward case uses can be easily obtained. For example, using Remark 2, a uniformly continuous NDEAT rate for $\dot{r}=1+e_{\sigma }$ can be found for the TT controller of the WMR presented in Section 34.4.2 of [1]. There, the authors use the kinematic model of a unicycle robot:

$$\left\{\begin{array}{l} \dot{x}=u_{1}\cos\theta \\ \dot{y}=u_{1}\sin\theta \\ \dot{\theta }=u_{2} \end{array}\right.\left\{\begin{array}{l} {\dot{x}}_{des}=u_{1,des}\cos\theta_{des}\\ {\dot{y}}_{des}=u_{1,des}\sin\theta_{des}\\ {\dot{\theta }}_{des}=u_{2,des} \end{array}\right.$$

where state **q** = (*x,y,θ*) represents the Cartesian coordinates of the driven wheel middle point and the orientation with respect to a fixed frame, and (*u_1_, u_2_*) the linear and angular speed of this point. Subscript ‘des’ is used for the virtual robot (the desired trajectory). They introduce the TT error definition **z^t^** *= h_e_*(**q***,***q_des_**(*t*)) = (*z_1_*, *z_2_*, *z_3_*) valid for $\theta -\theta_{des}\neq \pm \pi /2$ and given by

$$\left\{\begin{array}{l} {\dot{z}}_{1}=\left(x-x_{des}\right)\cos\theta_{des}+\left(y-y_{des}\right)\sin\theta_{des}\\ {\dot{z}}_{2}=-\left(x-x_{des}\right)\sin\theta_{des}+\left(y-y_{des}\right)\cos\theta_{des}\\ {\dot{z}}_{3}=\tan(\theta -\theta_{des}) \end{array}\right.$$

where dependence on *(t)* has been suppressed for desired coordinates for clarity reasons.

Additionally, through the definition of the Lyapunov function $V=\frac{1}{2}\left(z_{1}^{2}+z_{2}^{2}+\frac{1}{k_{2}}z_{3}^{2}\right)\;;\;k_{2}>0$, they propose a globally asymptotically stable TT control law that makes $\dot{V}$ negative semidefinite and uniformly continuous, provided that $\left|u_{1,des}\right|$ is uniformly continuous and does not tend to zero.

In order to apply NDEAT, gaining the benefits described previously, the necessary terms for Lemma 1 are calculated:

$${\left.\frac{\partial V}{\partial e}\right|}_{e=e^{r}}=\left(z_{1},z_{2},\frac{1}{k_{2}}z_{3}\right)\;,\;\frac{\partial h_{e}}{\partial q_{des}}f_{q,des}=\left(-u_{1,des}+z_{2}u_{2,des},z_{1}u_{2,des},-u_{2,des}\left(1+z_{3}^{2}\right)\right)$$

Finally, using Remark 2, an expression for the NDEAT tracking rate that preserves the validity of the same controller is obtained:

$$e_{\sigma }=-k_{\sigma }\left({\langle \left.\frac{\partial V}{\partial e}\right|}_{e=e^{r}},\frac{\partial h_{e}}{\partial q_{des}}f_{q,des}\rangle \right)=k_{\sigma }\left(z_{1}u_{1,des}+\frac{1}{k_{2}}z_{3}u_{2,des}\left(1+z_{3}^{2}\right)\right)\;;\;k_{\sigma }>0$$

With this $e_{\sigma }$, Lemma 1 ensures that the same control law is globally asymptotically stable for errors **z^r^***=h_e_*(**q***,***q_des_**(*r*)), and the convergence of the NDEAT tracking is, at least, as fast as that of TT. Note that if timing requirements were needed, the SDEAT method can be found in a similar manner.

Although several benefits of NDEAT tracking can be revealed for simple models, these benefits can be better observed for more complex robots including non-linearities. In the next section, EAT is compared with TT for a UAV, whose inputs saturate when their values surpass a certain bound.

## 3. Application of EAT for the PVTOL

Mobile robots, and more specifically UAVs, are a traditional application example when studying and selecting tracking methods because most of them do not need strict timing requirements. The interesting control problems associated with the vertical/short takeoff and landing aircraft has turned PVTOL into one of the most studied benchmarks for controller design. More concretely, the fact that PVTOL has non-minimum phase zero dynamics associated with its center of mass suggested that path following controllers could be more appropriate than tracking controllers [3].

Firstly, we recall in the next paragraphs the main equations for the PVTOL according to [26]. We refer the reader to this classic paper for further details on this system. Afterwards, the tracking method is transformed from TT to NDEAT and SDEAT by using the previous Lemmas. Finally, in the next section, several results are shown to demonstrate the benefits of using EAT methods instead of TT.

A simplified model for PVTOL is given by [26]:

(3) $$\begin{array}{l} m\ddot{x}=-\sin\theta \;T\\ m\ddot{y}=-mg+\cos\theta \;T\\ \ddot{\theta }=-\omega_{n}^{2}\sin\theta +k_{s}T\sigma_{2}\left(u_{2}\right)\\ \dot{T}=-k_{t}\left(T-\sigma_{1}\left(T_{d}\right)\right)\\ {\dot{T}}_{d}=u_{1} \end{array}$$

where *x, y* are the lateral and vertical positions, *θ* is the pitch angle, *T* is the actual propeller thrust in Newtons (which is controlled through a second order dynamics by the input *u_1_*, being *T_d_* the desired thrust), and input *u_2_* is the stabilator input used to generate a pitching movement. Constants in (3) are: *m* = 2.15 kg (mass of the aircraft), *g* = 4.98 ms^−2^ (effective gravity), $\omega_{n}=\sqrt{33}$ s^−1^ (natural frequency in pitch), *k_s_ = 5.4* kg^−1^m^−1^ (stabilator constant), and *k_t_ = 4* s^−1^ (thrust constant). Functions *σ* are saturations for the thrust and the stabilator inputs, with the following upper and lower limits: *max*(*σ_1_*) = 16, *min*(*σ_1_*) = 0, *max*(*σ_2_*) = 1, *min*(*σ_2_*) = −1.

It is well known that linearized forms of systems with nonholonomic constraints, such as WMRs (Wheeled Mobile Robots) and UAVs, are not controllable [27]. For this reason, other alternatives, such as feedback linearization have been profusely studied for these systems. In [26] it is shown that system (3) is feedback linearizable provided that saturations are not active. Using the linearizing coordinate change

(4) $$\left\{\begin{array}{l} x=\left(x,\dot{x},y,\dot{y},\theta ,\dot{\theta },T,T_{d}\right)\mapsto z\equiv \left(x,\dot{x},\ddot{x},\dddot{x},y,\dot{y},\ddot{y},\dddot{y}\right)\\ u=\left(u_{1},u_{2}\right)\mapsto v\equiv \left(x^{\left(4\right)},y^{\left(4\right)}\right) \end{array}\right.$$

the linearized dynamics (valid outside the saturation) result in

(5) $$\begin{array}{l} A_{o}=\left[\begin{array}{cccc} 0 & 1 & 0 & 0\\ 0 & 0 & 1 & 0\\ 0 & 0 & 0 & 1\\ 0 & 0 & 0 & 0 \end{array}\right]\;;\;B_{o}=\left[\begin{array}{c} 0\\ 0\\ 0\\ 1 \end{array}\right]\\ A=\mathrm{block}\;\mathrm{diagonal}\;\left\{A_{o},A_{o}\right\}\\ B=\mathrm{block}\;\mathrm{diagonal}\;\left\{B_{o},B_{o}\right\}\\ \dot{z}=Az+Bv \end{array}$$

A reference or desired path to be followed can be a feasible trajectory that fulfills (3). Therefore, it can be expressed as a function of a descriptor parameter *r*: **x_des_**(*r*), with desired inputs **u**_des_(*r*). Alternatively, for the linearized system, the desired path {**z_des_**(*r*), **v**_des_(*r*)} must fulfill $z_{des}^{’}=Az_{des}+Bv_{des}$.

Now we recall the control law for the linearized system (5) presented in [26], and we show that the same law can be used for EAT using the appropriate tracking rate (see Lemmas 1 and 2). Therefore, no design or tuning of a new controller is needed. A linear control law for system (5) is:

***v*** =***v***_*des*_ + ***K***(***z*** − ***z***_*des*_) (6)

where ***A_c_*** *= **A** + **BK*** is Hurwitz, which provides local stable trajectory tracking (global in (***z****, **v***) if there are no restrictions in these coordinates). Linear dynamics result in ${\dot{e}}_{z}=A_{c}e_{z}$, being ***e****_z_ = **z**-**z**_des_*. According to [26], the experience with the actual PVTOL shows that ***K*** can be decoupled for lateral and vertical modes and that values for ***K***: $K_{o}=\left[\begin{array}{cccc} -3604 & -2328 & -509.25 & -39 \end{array}\right]$, $K=\mathrm{block}\;\mathrm{diagonal}\left\{K_{o},K_{o}\right\}$ make the system perform properly. System input ***u*** can be calculated from ***v***, through (4) and (3). Thus, provided that {**x***(t)*, **u***(t)*} stays within the valid region, global exponential convergence to the desired trajectory (that is, to ***e****_z_* = 0) is guaranteed. Consequently, given a positive definite symmetric matrix ***Q*** *(**Q** > 0)*, there exists a unique positive definite symmetric matrix, ***P*** > 0, that fulfills the Lyapunov equation $A_{c}^{T}P+PA_{c}+Q=0$.

Let us first analyze the case of NDEAT. Using Lemma 1, it is evident that: if *a)* the same control (6) is applied, being $V=e_{z}^{T}Pe_{z}$ a Lyapunov candidate (from now on the superscript **^r^** of the error is omitted for simplicity reasons), and *b)* the chosen uniformly continuous NDEAT rate $\sigma =1+e_{\sigma }\left(e_{z},r\right)$ makes $-2e_{\sigma }\left({z^{\prime }}_{des}^{T}Pe_{z}\right)$ negative semidefinite with respect to ***e_z_***, then ***e_z_*** *= **z**-**z**_des_(r(t)) =* 0 is a globally asymptotically stable equilibrium point (if there are no restrictions in ***z, v*** coordinates).

The proof can be guided by using the change $\sigma =1+e_{\sigma }$, as in Lemma 1. In this case, the new linear dynamics for the same control law (6) is ${\dot{e}}_{z}=\dot{z}-\sigma \;z_{des}^{’}=A_{c}e_{z}-e_{\sigma }z_{des}^{’}$. By computing the derivative of *V*, and using the new linear dynamics, we obtain

(7) $$\dot{V}=e_{z}^{T}\left(A_{c}^{T}P+PA_{c}\right)e_{z}-2e_{\sigma }\left({z^{\prime }}_{des}^{T}Pe_{z}\right)$$

Using the hypothesis and the Lyapunov equation, it holds that $\dot{V}=-e_{z}^{T}Qe_{z}-2e_{\sigma }\left({z^{\prime }}_{des}^{T}Pe_{z}\right)$ is negative definite and uniformly continuous in time.

An evident proposal that makes the last term of (7) quadratic is:

(8) $$\sigma =1+2K_{\sigma }\left({z^{\prime }}_{des}^{T}Pe_{z}\right)=1+2K_{\sigma }z_{des}^{’},e_{z}{}_{P},\;K_{\sigma }>0$$

but other functional proposals for σ are possible (depending on the application tracking requirements).

**Remark** **5.** *The proposed form (8) of* σ *includes a dot product, which gives an idea of the relative posture (the “sign” of the errors) of the real and virtual robots when the robot is not too “far” (according to the distance function given by matrix **P**) from the reference point. When the robot is “ahead” with respect to **z_des_**(r) (along the direction specified by **z** ¢**_des_**(r)), then this dot product will be positive, but when the robot is delayed, it will be negative. If this product is zero, the errors are perpendicular to the desired velocities, and the robot is neither ahead nor delayed. Therefore, the tracking rate can be greater or less than 1. When the robot is ahead (according to the previous dot product criterion), it is intuitive that a faster rate will get the reference point closer to the robot. On the other hand, for a delayed robot, the lower value of* σ *means that the desired point will “wait” for the robot.*

If the SDEAT method were to be applied, another Lyapunov function can be defined according to Lemma 2. Using $V_{2}=e_{z}^{T}Pe_{z}+\frac{1}{2}A_{rt}{\left(r-t\right)}^{2}$ and proceeding correspondingly, (7) results in:

$${\dot{V}}_{2}=e_{z}^{T}\left(A_{c}^{T}P+PA_{c}\right)e_{z}-e_{\sigma }\left(2{z^{\prime }}_{des}^{T}Pe_{z}-A_{rt}\left(r-t\right)\right)$$

An evident SDEAT proposal that keeps ${\dot{V}}_{2}$ uniformly continuous in time and negative definite (other σ are possible), is to make the last term quadratic by carrying out

(9) $$\sigma =1+K_{\sigma }\left(2z_{des}^{’},e_{z}{}_{P}-A_{rt}\left(r-t\right)\right),\;K_{\sigma }>0$$

Therefore, if *a)* the same control (6) is applied and *b)* the uniformly continuous EAT rate $\sigma =1+e_{\sigma }$ makes $-2e_{\sigma }\left({z^{\prime }}_{des}^{T}Pe_{z}\right)-A_{rt}\left(r-t\right)$ negative definite, then ***e_z_*** = ***0*** is a global asymptotically stable equilibrium point. The proof can be guided in a way similar to that of the proof of Lemma 2.

## 4. Simulation Results

In this section, simulation results comparing the TT and EAT behaviors for the previous UAV system are presented and discussed. Although the results are shown for this system, these analyses and conclusions can be extended to other systems for which a classic control law was previously obtained. Advancing one of the conclusions of our results, using numerically the same control law for EAT leads to a faster and much more robust convergence as Remark 1 points out. The reason is that errors that affect the tracking are partially “absorbed” by the tracking rate $\dot{r}$. Therefore, a final thought is worth mentioning: for complex systems, it is more difficult to find robust control laws that behave well enough under several problems. When this happens, the EAT method may provide a form of avoiding oscillations, divergences, error enlargements, etc.

Two tests are to be analyzed using a SIMULINK/MATLAB model [28]: a) the diverse initial extreme conditions, and b) the convergence to a desired path that cannot be executed by the PF presented in [26] because the whole path fulfills the projection used there. The first test is focused on big errors because in the case of small errors the simulated system’s behavior will be similar to that of an exponential convergent system. The second test is intended to confirm the advantage of EAT versus PF, which is the validity of EAT for all kinds of paths.

The chosen form of σ for NDEAT is that of (8) with *K_s_* = 0.0010 m^−1^s^−1^. To conduct a fair comparison between NDEAT and SDEAT methods, the constant *K_s_* of SDEAT is the same as that of NDEAT, with *A_rt_* = 400 m^2^/s^2^. Greater values of *A_rt_* will bring the tracking closer to that of TT, while lower ones will bring it closer to that of NDEAT. An important property of σ is the linearity around ***e_z_*** = 0.

As our intention is to show the benefits of EAT against TT for a same controller, we have used exactly the same controller of [26]. Therefore, the Lyapunov equation matrices used are ***Q = I***, and by solving Lyapunov equation: ***P*** = block diagonal {***P_o_****,**P_o_***}, with

$$P_{0}=\left[\begin{array}{l} \;436.3905\;281.1773\;53.4184\;0.0001\\ \;281.1773\;189.7791\;39.2207\;0.1210\\ \;53.4184\;39.2207\;10.4683\;0.0780\\ \;0.0001\;0.1210\;0.0780\;0.0148 \end{array}\right]$$

**Figure 4:**
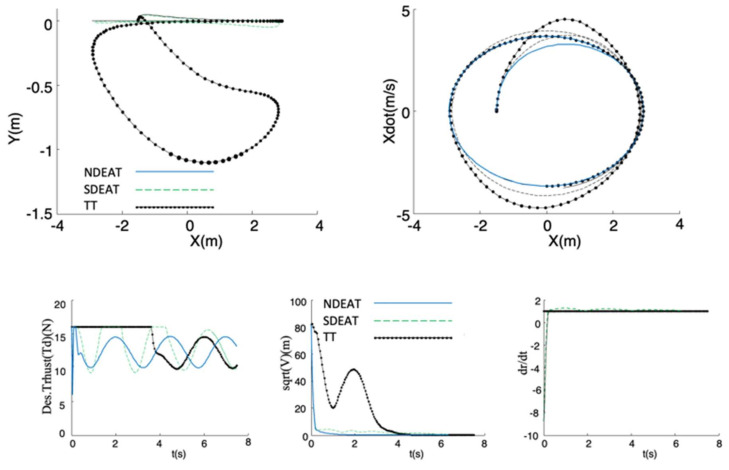
Comparison of NDEAT, SDEAT, and TT when the robot is delayed (desired path consists on a periodic horizontal motion *x_des_(r) = A sin(w_ref_ r)*, *y_des_(r) = constant*). **Up**: **Left**, *Y/X* trajectories; **Right**: evolution of $\dot{X}/X$ **Bottom**: **Left**, Desired Thrust *T_d_* (with saturation). **Middle**: Lyapunov function versus time. **Right**: evolution of σ=dr/dt with respect to time.

**Figure 5:**
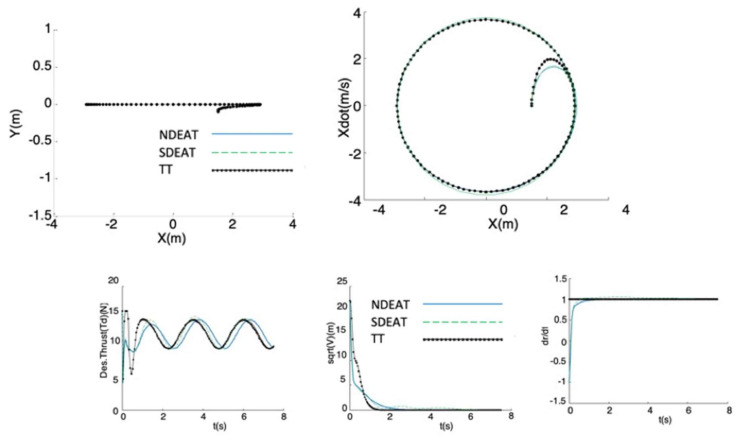
Comparison of NDEAT, SDEAT and TT when the robot is ahead (same desired path as previous Figure 4). **Up**: **Left**, Y/X trajectories; **Right**: evolution of $\dot{X}/X$ **Bottom**: **Left,** Desired Thrust Td (with saturation). **Middle**: Lyapunov function versus time. **Right**: evolution of σ=dr/dt with respect to time.

**Figure 6:**
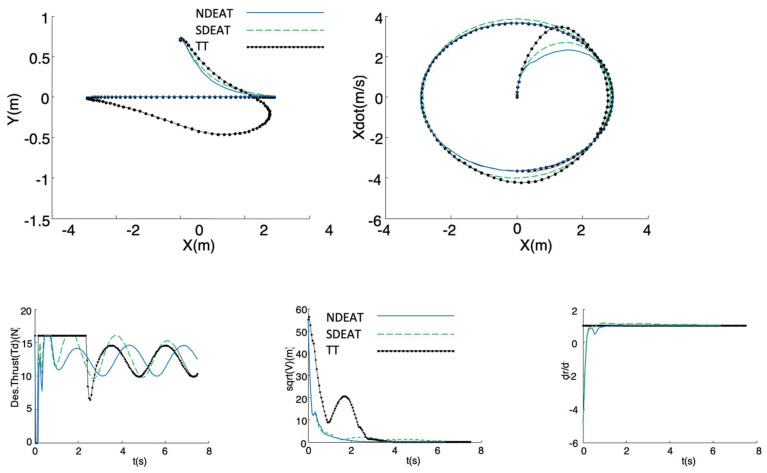
Comparison of NDEAT, SDEAT and TT when the robot is neither delayed nor ahead. **Up**: **Left**, Y/X trajectories (same desired path as previous Figure 4); **Right**: evolution of $\dot{X}/X$ **Bottom**: **Left**, Desired Thrust Td (with saturation). **Middle**: Lyapunov function versus time. **Right**: evolution of σ=dr/dt with respect to time.

The first test (Figure 4, Figure 5 and Figure 6) analyzes the tracking of a periodic lateral motion *x_des_(r) = A sin(w_ref_ r)* with constant *y_des_(r) =* 0, where *w_ref_* = 2π/5 s^−1^, and *A* = 1.857 π/2 m. The initial desired state and value of parameter *r* are: **z_des_** = 0; *r* = 0, for all tests. Two considerable initial position errors in *x* and *y* are tested in order to compare EAT with TT (see Table 1, where the corresponding figures are also shown). The rest of the real initial states are the same as those desired.

The experiment is delayed for only 7.5 s, in order to see the transients more clearly. For all figures, NDEAT curves are drawn with solid lines, SDEAT with dashed lines and TT with dash-dotted lines.

For all the tests, it can be observed that the method with the fastest and best convergence to the path is NDEAT. According to Remark 1, NDEAT and SDEAT convergences are obviously faster than that of TT. This fact can be also verified by several reasons. First, Lyapunov function diminution is very much faster for EAT than for TT (especially when the robot is delayed). Moreover, the Lyapunov function is not decreasing when the system comes into the saturation zone (*T_d_* > 16 *N*), which occurs in many occasions and for long periods when using TT. This zone should be avoided because when the system enters this zone, the feedback linearization control is not valid. As a consequence, PVTOL control is lost and in some occasions coordinate *Y* goes so low that the actual system may collide with the ground (a value below −0.5 m is not possible for the real PVTOL). In any case, the evolution of *s* is the expected one: during the first transients, it reaches values that are far from one, in order to “look for” the best desired reference (which fulfills that $z_{des}^{’},e_{z}{}_{P}$ is near zero). Afterwards, it remains near one for NDEAT but greater than one for SDEAT, which is necessary in order to reduce the difference between *r* and *t*. After 7 s, parameter *r* has almost reached *t* in SDEAT; on the contrary, a gap remains when NDEAT is applied.

Other interesting points are the following: when the robot is ahead, all tracking methods behave satisfactorily. Since the actual system response is slow (mainly due to the second order dynamics of input *u*_1_) this case is not as critical as the delay in the robot’s posture. Nevertheless, TT is the only method that comes into saturation in the first moments of this test. Finally, TT shows also significant problems for the last trial (the robot is neither ahead nor delayed). Although coordinate *X* presents a small error, *Y* falls considerably. This is because the TT reference posture is continuously increasing, which implies high input values that take the system out of the linear zone. In conclusion, one of the strongest points of NDEAT is that its behavior is almost the same as the PF approach found in [26]. This can be observed very patently when the robot is delayed.

**Figure 7:**
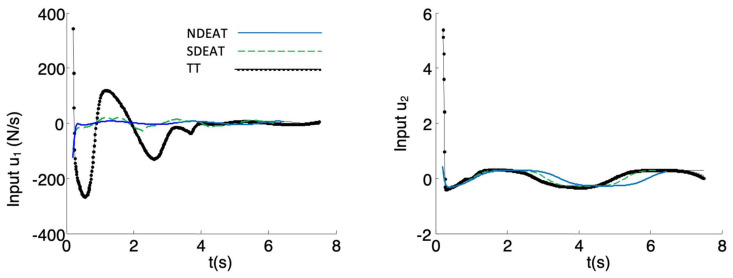
Inputs *u* when the robot is delayed. During transients, NDEAT demands less input than SDEAT, and SDEAT demands much less than TT. The first instants are not depicted because TT inputs rise to very high values, which would reduce the scale of the plot too much.

Moreover, and due to its large errors, the real speeds demanded by TT are greater than those of NDEAT (Figure 7). It is obvious that input limitations will further degrade TT’s response. In the end, the TT method introduces more oscillations than EAT, and it has a transient response that separates the robot from the desired path. This is a well-known advantage of PF that EAT retains [1]. Concretely, for the PVTOL system the states variables barely enter the saturation zone when applying NDEAT. Moreover, if time determinism were needed, SDEAT would be a possible option, which avoids TT drawbacks and maintains the system’s response near to that of NDEAT.

**Figure 8:**
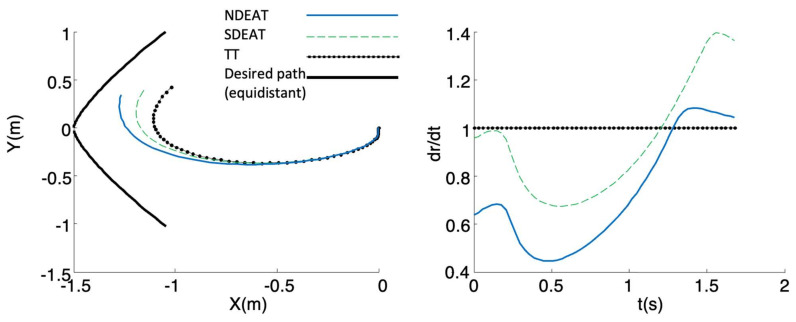
NDEAT, SDEAT and TT when the path is equidistant to the origin. **Left**: XY desired and real trajectories. **Right**: evolution of σ=dr/dt with respect to time.

In the second test, the segment that fulfills the PF projection used in [26] is previously computed to serve as the path for the tracking (thick line in Figure 8). This path is composed of the points that are equidistant from the origin according to the projection. The initial posture of the robot is the origin (**x** = 0), and the desired initial position is *(X,Y)* = (−1.5,0). As can be seen, there is no racking problem for any EAT method. Moreover, as expected, NDEAT converges slightly better than SDEAT, and SDEAT converges better than TT. Note that no comparison with PF is possible because the projection is not defined for it. Moreover, and due to its large errors, the real speeds demanded by TT are greater than those of NDEAT. It is obvious that speed limitations will degrade TT’s response even more. In the end, the TT method introduces more oscillations than EAT, and it has a transient response that separates the robot from the desired path. This is also another advantage of PF [1] that EAT also retains.

Similar comparisons can be made for other perturbations, such as saturation of inputs, path’s curvature discontinuities, introduction of a scale between the inputs demanded by the control and the real ones, etc. On the whole, this is because the inputs demanded by TT are usually greater than those asked by EAT (see Figure 7), so TT experiences more problems in the tracking. Thus, the EAT method is more robust than TT against perturbations or unmodeled dynamics because the adaptive variation of *r* facilitates robustness. Finally, it is important to observe that the qualitative behavior of EAT is similar to that of PF, i.e., $\dot{r}$ is reduced in the presence of large errors until the system approaches the path. Obviously, both methods are constructed in a very different way, so it is not easy to make a quantitative comparison.

## 5. Conclusions

In this work, it is illustrated how a Lyapunov-based trajectory tracking control law can be used for error adaptive tracking methods for any system. This is carried out by selecting the proper rate for the progression of the descriptor parameter of the reference curve. This way, the burden of finding a new controller is not necessary. When EAT method is applied to a UAV (PVTOL), it is shown, through several tests, that error adaptive tracking methods outperform trajectory tracking ones using exactly the same controller (with identical parameters). We can conclude that the behavior of the several alternatives of error adaptive tracking is much better than that of a trajectory tracking under large errors, disturbances, unmodeled parameters or delayed response. This is because in error adaptive tracking, pace adapts to system errors. Two additional advantages of error adaptive tracking are also presented: a) it conserves most of the advantages of the path following method, and b) it avoids one of the main drawbacks of the path following method, that is, it is valid for all feasible trajectories. Finally, the benefits of the variant here called “soft deterministic error adaptive tracking” are also illustrated. This alternative presents almost the same excellent behavior as any error adaptive tracking when errors are large because temporal determinism is ignored in these situations. However, once errors have decreased, it gains the additional benefit of taking into account timing determinism as in classic trajectory tracking.

## Figures and Tables

**Table 1:** Initial robot positions and their corresponding figures.

Figure	*x-xdes* (m)	*y-ydes* (m)	Case
Figure 4	−1.5	0	robot is delayed
Figure 5	1.5	−0.1	robot is ahead
Figure 6	0	0.7	robot is neither ahead nor delayed
